# Management of patients with lymphoma and COVID‐19: Narrative review and evidence‐based practical recommendations

**DOI:** 10.1002/hon.3086

**Published:** 2022-10-25

**Authors:** Francesco Passamonti, Emanuele Nicastri, Alice Di Rocco, Attilio Guarini, Adalberto Ibatici, Stefano Luminari, Malgorzata Mikulska, Carlo Visco

**Affiliations:** ^1^ Department of Medicine and Surgery University of Insubria Varese Italy; ^2^ Hematology, ASST Sette Laghi, Ospedale di Circolo Varese Italy; ^3^ National Institute of Infectious Diseases “L. Spallanzani” IRCCS Roma Italy; ^4^ Department of Cellular Biotechnologies and Hematology Hematology Unit Sapienza University Roma Italy; ^5^ Hematology Unit IRCCS Istituto Tumori “Giovanni Paolo II” Bari Italy; ^6^ Hematology Unit and Bone Marrow Transplantation IRCCS Ospedale Policlinico San Martino Genova Italy; ^7^ Hematology Unit Azienda Unità Sanitaria Locale, IRCCS Reggio Emilia Reggio Emilia Italy; ^8^ Dipartimento CHIMOMO Università di Modena e Reggio Emilia Reggio Emilia Italy; ^9^ IRCCS Ospedale Policlinico San Martino Genova Italy; ^10^ Division of Infectious Diseases Department of Health Sciences (DISSAL) University of Genova Genova Italy; ^11^ Department of Medicine Section of Hematology University of Verona Verona Italy

**Keywords:** antiviral, COVID‐19, immunosuppression, lymphoma, monoclonal antibody, SARS‐CoV‐2

## Abstract

Patients with hematologic malignancies can be immunocompromized because of their disease, anti‐cancer therapy, and concomitant immunosuppressive treatment. Furthermore, these patients are usually older than 60 years and have comorbidities. For all these reasons they are highly vulnerable to infection with severe acute respiratory syndrome coronavirus‐2 (SARS‐CoV‐2) and have an increased risk of developing severe/critical Coronavirus disease 2019 (COVID‐19) compared to the general population. Although COVID‐19 vaccination has proven effective in reducing the incidence of severe/critical disease, vaccinated patients with lymphoma may not be protected as they often fail to develop a sufficient antiviral immune response. There is therefore an urgent need to address the management of patients with lymphoma and COVID‐19 in the setting of the ongoing pandemic. Passive immunization with monoclonal antibodies against SARS‐CoV‐2 is a currently available complementary drug strategy to active vaccination for lymphoma patients, while monoclonal antibodies and antiviral drugs (remdesivir, ritonavir‐boosted nirmatrelvir, and molnupiravir) have proven effective in preventing the progression to severe/critical COVID‐19. In this narrative review we present the most recent data documenting the characteristics and outcomes of patients with concomitant lymphoma and COVID‐19. Our ultimate goal is to provide practice‐oriented guidance in the management of these vulnerable patients from diagnosis to treatment and follow‐up of lymphoma. To this purpose, we will first provide an overview of the main data concerning prognostic factors and fatality rate of lymphoma patients who develop COVID‐19; the outcomes of COVID‐19 vaccination will also be addressed. We will then discuss current COVID‐19 prophylaxis and treatment options for lymphoma patients. Finally, based on the literature and our multidisciplinary experience, we will summarize a set of indications on how to manage patients with lymphoma according to COVID‐19 exposure, level of disease severity and former history of infection, as typically encountered in clinical practice.

## 1 INTRODUCTION

Coronavirus disease 2019 (COVID‐19) is classified by the World Health Organization (WHO) into four severity degrees: mild, moderate, severe, and critical.^1^ Patients infected with the causative virus, severe acute respiratory syndrome coronavirus‐2 (SARS‐CoV‐2), that develop critical disease are characterized by respiratory failure, acute respiratory distress syndrome, septic shock, or multiorgan dysfunction or failure.^1^ A number of risk factors associated with increased COVID‐19‐related morbidity and mortality have been identified, including age >60 years, male gender, and underlying comorbidities, namely diabetes, hypertension, cardiac disease, chronic lung disease, cerebrovascular disease, chronic kidney disease, immunosuppression, obesity, and cancer.^1^, ^2^

Since the outbreak of the COVID‐19 pandemic, epidemiological studies worldwide have shown that cancer patients are highly vulnerable to SARS‐CoV‐2 infection and may be at risk for severe COVID‐19.^3^, ^4^, ^5^, ^6^, ^7^, ^8^, ^9^, ^10^ Patients with hematologic malignancies appear to have worse COVID‐19‐related outcomes than those with solid malignancies, but this point has not been conclusively established.^8^, ^11^, ^12^ Cancer patients are a vulnerable group for several reasons. They can be immunocompromized because of their disease, anti‐cancer therapy, and concomitant immunosuppressive treatment. Furthermore, a large proportion of them are aged >60 years and have comorbidities.^5^ With regard to the role of immunosuppression, it should be noted that an attenuated immune system may in fact protect patients against multi‐organ injury caused by the excessive inflammatory response that characterizes severe/critical COVID‐19.^13^, ^14^

Lymphomas are a heterogeneous group of malignant neoplasms of lymphocytes that can affect the lymphatic tissue, bone marrow, and any other body organ.^15^, ^16^ Traditionally, they are divided into Hodgkin lymphomas (HL) and non‐Hodgkin lymphomas (NHL), with the latter accounting for approximately 90% of all lymphomas.^15^ NHL are often treated with chemotherapy with or without the addition of monoclonal antibodies against CD20‐positive B lymphocytes, inducers of T lymphocyte depletion, or immunomodulators. As seen for other diseases, the COVID‐19 pandemic has introduced significant changes in oncologic practice, with a substantial burden on patients and health care providers and the potential worsening of patient outcomes.^8^ In addition, although COVID‐19 vaccination has proven effective in reducing the incidence of severe COVID‐19 in the general population,^17^, ^18^, ^19^, ^20^ vaccinated patients with lymphoma may not be protected as they often fail to develop a sufficient antiviral immune response.^21^, ^22^, ^23^ Also, as we have learned from the omicron variant, new SARS‐CoV‐2 strains may be only partially neutralized by existing vaccines.^21^ Lymphoma patients are therefore at high risk of breakthrough SARS‐CoV‐2 infection and indications on how to manage this vulnerable group are urgently needed.^24^ Alternative prophylactic strategies, including passive immunization with monoclonal antibodies to the spike protein of SARS‐CoV‐2,^25^, ^26^, ^27^, ^28^, ^29^, ^30^ and treatment of mild or moderate COVID‐19 with antiviral agents^31^, ^32^, ^33^ need to be explored in lymphoma patients. In addition, programs of booster vaccinations need to be implemented as the emerging data on additional vaccine doses in patients with no seroconversion after the first vaccination cycle are promising.^34^

In this narrative review we present the most recent data documenting the characteristics and outcomes of patients with concomitant lymphoma and COVID‐19; our objective is to provide evidence‐based guidance in the management of these vulnerable patients from diagnosis to treatment and follow‐up. To this purpose, we will first report the main data concerning prognostic factors and mortality rates of lymphoma patients who develop COVID‐19; the outcomes of COVID‐19 vaccination will also be addressed. We will then discuss current treatment options for SARS‐CoV‐2‐infected subjects at high risk of progressing to severe/critical disease. Finally, based on the literature and our multidisciplinary experience, we will provide practical guidance on how to manage patients with lymphoma in the setting of ongoing COVID‐19 pandemic.

## 2 METHODS

We searched PubMed with the terms “lymphoma AND (COVID‐19 OR SARS‐CoV‐2)” for any type of article published in English up to 27 May 2022. Retrieved articles were screened based on their title and abstracts. Articles of potential interest were further selected by giving the preference to well‐designed studies, large patient populations, and systematic reviews or meta‐analyses of the literature. Additional literature was retrieved from the reference list of articles identified in the PubMed search.

## 3 OUTCOMES OF LYMPHOMA PATIENTS WITH COVID‐19

### 3.1 Characteristics of patients who develop severe or critical disease

The literature reporting specifically on patients with lymphoma who developed COVID‐19 is limited.^35^, ^36^, ^37^, ^38^ The studies attempting to define the epidemiology of COVID‐19 in lymphoma, or in hematologic malignancies, were mostly performed during the first months following the COVID‐19 pandemic outbreak and involved therefore unvaccinated individuals who developed severe/critical disease; observation times ranged from 1 to a few months. Table 1 summarizes the most relevant information emerging from these early observations in patients with lymphoma and other hematologic malignancies. Overall, patients were predominantly male, were aged over 55 years, and presented relevant comorbidities. The majority had severe/critical COVID‐19. Most patients had been in treatment for their lymphoma during the 12 months preceding the diagnosis of COVID‐19. They presented with various lymphoma statutes and approximately half of them were in remission.^35^, ^37^

**TABLE 1:** COVID‐19 in patients with lymphoma and other hematologic malignancies

Study	Design	Demographic and clinical characteristics	Mortality rate	Predictors of mortality
Passamonti et al., 2020^6^	Retrospective, multicenter study Observation period: 3 m To determine mortality and predictive factors of mortality In‐patients with HM, *N* = 536 (44% with lymphomas)	Median age 68 (58–77) yr 63% male Median CCI 4 (3–6) 50% with severe/critical COVID‐19	37%	Older age Progressive HM (HR 2.10, 95% CI 1.41–3.12) HM type (indolent lymphoma HR 2.19, 95% CI 1.07–4.48; aggressive lymphoma, HR 2.56, 95% CI 1.34–4.89) Severe/critical COVID‐19 (HR 4.08, 95% CI 2.73–6.09)
Yigenoglu et al., 2020^7^	Retrospective analysis To compare the outcomes of pts with HM, *N* = 740 (33.7% with lymphomas) versus pts with no cancer (*N* = 740)	Median age: 56 versus 56 y Male gender: 53.6% versus 54.1% 28.7% versus 19.6% with severe/critical COVID‐19	CFR 13.8% versus 6.8%, *p* = 0.0001; 10.8% for NHL and 14.8% for HL Significantly worse COVID‐19 outcomes for HM pts versus pts with no cancer	NA
Garcia‐Suarez et al., 2020^39^	Prospective, registry study To define mortality rate, prognostic factors In‐ and out‐patients with HM, *N* = 697 (69% with lymphoid malignancy)	Median age 72 (IQR 60–79) yr 60% male 59% on active treatment for HM 62% with severe/critical COVID‐19	33%	Age ≥60 yr >2 comorbidities AML versus NHL Lymphoma treatment with mAb versus no treatment
Lamure et al., 2020^35^	Retrospective, multicenter study To characterize presentation and outcomes Pts with lymphoma hospitalized for COVID‐19, *N* = 89	Median age, 67 (19–92) yr 66% male 72% with comorbidities 44% with lymphoma complete remission	34% 30‐d OS, 71% (95% CI 62%–81%)	Age ≥70 yr (HR 2.87, 95% CI 1.20–6.85, *p* = 0.02) Relapsed/refractory lymphoma (HR 2.54, 95% CI 1.14–5.66, *p* = 0.02)
Regalado‐Artamundi et al., 2021^36^	Retrospective registry study To define epidemiology and predictors of death In‐ and out‐patients with lymphoma, *N* = 177	Median age 70 (IQR 56–77) yr 55.9% male >70% with comorbidities 49.7% on active treatment for lymphoma 86.3% hospitalized due to COVID‐19	34.5%	Age > 70 yr Comorbidities Active disease (vs. complete response, HR 2.770 [95% CI 1.143–6.712, *p* = 0.024])
Duléry et al., 2021^40^	Retrospective, multicenter study To examine prolonged length of stay in hospital and its determinants 111 lymphoma in‐pts	Median age, 65 y 71% treated for lymphoma within 1 yr 12% with relapsed/refractory lymphoma	6‐m survival 69%	Recent anti‐CD20 therapy was associated with prolonged stay in hospital and higher risk of death (HR 2.17, 95% CI 1.04–4.52, *p* = 0.039)
Pagano et al., 2021^41^	EPICOVIDHEA registry survey To characterize epidemiology and predictors of mortality Pts with HM, *N* = 3801; *N* = 1084 with NHL, *N* = 135 with HL	Median age 65 (54–74) yr 58.5% male 30.8% with complete remission of HM; 51.6% with active HM 63.8% with severe/critical COVID‐19	Overall 31.2%; COVID‐19‐related, 22.2%	Age Active HM Chronic cardiac disease Renal impairment Smoking history ICU stay
Visco et al., 2022^37^	Multicenter retrospective study and analysis of prospectively collected data To identify predictors of death In‐ and out‐patients with lymphoma, *N* = 856^a^ (*N* = 468 in‐pts, *N* = 388 out‐pts)	Median age, 63 (19–94) yr 59% male 46% with complete remission of lymphoma 59% of hospitalized pts had severe/critical COVID‐19 versus 8% of out‐pts	Overall, 19.5% 33.4% for in‐pts 3.8% for out‐pts	Age > 65 yr Male gender ALC < 650 × 10^9^/L Platelets < 100 × 10^9^/L

Abbreviations: ALC, absolute lymphocyte count; CCI, Charlson Comorbidity Index; CFR, case fatality rate; HL, Hodgkin lymphoma; HM, hematologic malignancy; HR, hazard ratio; ICU, intensive care unit; NHL, non‐Hodgkin lymphoma.

^a^ 237 patients included also in the study by Passamonti et al.

### 3.2 Case fatality ratio and prognostic factors

Reported overall mortality rates of patients with lymphoma and COVID‐19 were consistently elevated (>30%) compared to those of the general population with COVID‐19 and lymphoma patients without COVID‐19 (Table 1).^35^, ^36^, ^37^ In the study by Visco et al., most deaths (91%) were related to COVID‐19.^37^ The 30‐day mortality rates for mild, severe, and critical COVID‐19 were, 4%, 22%, and 45%, respectively; the corresponding 100‐day rates were 9%, 38%, and 75%, respectively.^37^ The EPICOVIDHEA survey had an extended observation period that included the first (March–May 2020) and the second (October‐December 2020) pandemic waves.^41^ According to the survey, the overall mortality decreased from 40.7% during the first wave to 24.8% during the second wave (*p* < 0.0001).^41^ Following characteristics were consistently identified as predictors of poor outcomes for patients with concomitant lymphoma and COVID‐19: older age, presence of relevant comorbidities, active malignancy (Table 1). Lamure et al. noted that, in the absence of the risk factors older age and refractory lymphoma, the mortality of the study cohort was similar to that of the general French COVID‐19 population.^35^

Based on the four parameters identified by multivariable analysis as the strongest predictors of mortality (age >65 years, male gender, lymphopenia, and thrombocytopenia), Visco et al. designed a predictive model for survival, to identify both in‐ and out‐patients at increased risk of death during the initial 2 months following COVID‐19 diagnosis.^37^ The model assigns a total score from 0 to 5 based on the presence or absence of the four variables, and classifies risk into three levels, low (total score 0–1), intermediate (2–3), and high (4–5). As the model uses easily available variables, its implementation in clinical practice should be straightforward.

With regard to the effects of antineoplastic treatment on patient outcomes, the various therapies used at the time of COVID‐19 diagnosis, or in the few months prior to diagnosis, did not seem to have major consequences on disease course and mortality. Passamonti et al. investigated COVID‐19 mortality according to antineoplastic therapy taken at COVID‐19 onset or in the previous 3 months and did not find an increased risk of mortality.^42^ In patients with follicular lymphoma on maintenance with anti‐CD20 therapy following remission, a lower mortality (31%) was reported compared to chemotherapy plus anti‐CD20 therapy (47%), or chemotherapy alone (44%).^6^ Lamure et al. found no association between anti‐CD20 therapy and increased mortality; an increase in mortality was seen with bendamustine in the high‐risk group of patients with relapsed/refractory lymphoma.^35^ Regalado‐Artamendi investigated whether the persistence of SARS‐CoV‐2 infection was related to antineoplastic therapy, anti‐CD20 therapy in particular; 6 months after the onset of infection, the proportion of patients treated with anti‐CD20 therapy was similar between the group with a negative SARS‐CoV‐2 test and the group with a persistently positive test.^36^ In the lymphoma population analyzed by Visco and colleagues, 33% had recent treatment (≤6 months) and 24% had been treated >6 months before COVID‐19 diagnosis.^37^ These treatments, including anti‐CD20 therapy and bendamustine, did not appear to worsen mortality rates. Based on these preliminary findings, a common notion is that discontinuing effective antineoplastic treatment may not be justified in lymphoma patients in the pandemic.^6^ This notion is reflected in the current guidelines for the treatment of patients with hematological malignancies during the COVID‐19 pandemic.^24^ It should be noted, however, that the impact of previous antineoplastic therapy on COVID‐19 outcomes still needs to be fully understood, as evidence of a negative effect has been reported, for example, in the study by Dulery et al. examining the reason(s) determining a prolonged stay in hospital (Table 1).^40^

### 3.3 COVID‐19 course in vaccinated lymphoma patients

Data documenting the outcomes of patients with lymphoma who have been vaccinated against SARS‐CoV‐2 are lacking. Evidence from studies in cancer patients suggests that those who develop COVID‐19 following full vaccination continue to be at risk of substantial comorbidity and death.^43^, ^44^ Risk factors for developing COVID‐19 following vaccination include older age, single vaccine dose without previous COVID‐19, and anti‐CD20 therapy in the previous 3 months.^44^ In January 2021, the EPICOVIDEHA registry started to collect prospectively the data of patients with hematologic malignancies who had been vaccinated against COVID‐19.^45^ The preliminary data of 113 patients who developed COVID‐19 following partial or complete vaccination have shown that the majority of these patients had lymphoproliferative malignancies (>80%), were male (61.1%) and were aged >50 years (85.8%).^45^ Almost 70% of them were on anticancer treatment when diagnosed with COVID‐19 or within the prior 3 months. COVID‐19 was severe/critical in 60.4% of patients; the overall mortality at 30 days was 12.4%, with COVID‐19 being the primary or secondary cause in 13 of the 14 patients who died. A recent prospective study of 365 patients with hematologic malignancies who had completed the first cycle of anti‐SARS‐CoV‐2 vaccination compared the clinical characteristics of COVID‐19 developed before or after vaccination.^34^ The study found that in vaccinated individuals the rates of critical disease (10% vs. 33%, *p* = 0.0242) and hospitalization (17% vs. 50%, *p* = 0.0024) were significantly lower and the median disease duration was significantly shorter (16 vs. 22 days, *p* = 0.0094) than in unvaccinated individuals.^34^

Overall, these preliminary results suggest that vaccination reduces the mortality rate compared to the pre‐vaccination period. However, they also show that patients with hematologic malignancies continue to be vulnerable.

### 3.4 Long‐term consequences of COVID‐19

It is increasingly recognized that a proportion of patients who have survived COVID‐19 experience long‐term sequelae of the disease, of variable severity and affecting different organs. A recent retrospective analysis of OnCovid, a European registry enrolling patients with a history of solid or hematologic cancer who were diagnosed with SARS‐CoV‐2 infection, showed that COVID‐19 long‐term complications may affect also cancer patients, with a negative impact on recovery from COVID‐19 and oncologic outcomes.^46^ The most frequently reported sequelae included respiratory symptoms, residual fatigue, weight loss, and neurocognitive symptoms. The rate of sequelae in the subgroup of patients with hematological malignancies (13.3%) was similar to that of the overall cancer population of the study (15.0%). The study also showed that COVID‐19 long‐term sequelae were associated with shorter survival and a greater likelihood to discontinue antineoplastic therapy.

## 4 IMMUNIZATION OF LYMPHOMA PATIENTS AGAINST SARS‐CoV‐2

### 4.1 Immunization following SARS‐CoV‐2 infection

Humoral responses to SARS‐CoV‐2 infection have been shown to be less pronounced and slower in individuals with hematologic conditions compared to the general population.^23^ In a retrospective study within the ITA‐HEMA‐COV project evaluating the humoral immune response to SARS‐CoV‐2 in 237 patients with hematologic malignancies (51.1% with lymphoid neoplasms) who had been exposed to SARS‐CoV‐2, 31% of patients were serologically negative.^42^ Chemoimmunotherapy was significantly associated with lower rates of seroconversion (OR 3.42, 95% CI 1.04–11.21; *p* = 0.04). Notably, this association persisted up to 6 months following therapy discontinuation. Based on these observations and on the experience with other vaccines,^47^ it can be expected that many patients with lymphoma will fail to mount an adequate response to COVID‐19.

### 4.2 Vaccination

Growing evidence from studies of COVID‐19 vaccination shows that patients with hematologic malignancies, and especially those on antineoplastic treatment, have indeed an attenuated humoral response.^21^, ^23^, ^48^, ^49^, ^50^, ^51^, ^52^, ^53^, ^54^, ^55^ An observational study in 67 patients with lymphoma and 35 healthy controls who received COVID‐19 mRNA vaccines compared the titers of IgG to the SARS‐CoV‐2 spike protein and found that a substantial proportion of lymphoma patients failed to respond, while all controls responded.^50^ Current lymphoma treatment (especially B‐cell depleting therapies), or treatment within the previous 2 years, were associated with a poor or no vaccine response. Notably, treatment‐naïve lymphoma patients, or patients who had discontinued treatment for >2 years were found to respond to vaccination, similarly to the control group.^50^ Similar results were reported in an interim analysis of the UK PROSECO study designed to evaluate responses to COVID‐19 vaccination in lymphoid malignancies.^51^ Vaccinated lymphoma patients on treatment (i.e., receiving anti‐lymphoma treatment at the time of first vaccine dose administration, having completed treatment ≤6 months before, or starting treatment <1 month after the first vaccine dose) had significantly lower IgG titers than healthy controls and lymphoma patients not on treatment. The responses of lymphoma patients not on treatment were comparable to those of the healthy controls. The authors pointed out that lymphoma patients vaccinated while on anti‐CD20 therapy should be revaccinated 6 months after treatment completion.^51^ A study attempted to define the factors that allowed lymphoma patients to achieve a good humoral response after two injections of the BNT162b2 mRNA vaccine (cutoff for positive response set at 50 AU/ml).^23^ Half of the 162 lymphoma patients enrolled had a positive humoral response. The time from the discontinuation of anti‐CD20 therapy to vaccination was found to be a robust predictive factor of the IgG antibody titers, with longer times being associated with higher antibody titers.

Finally, antibody response (seroconversion rate) of patients with hematologic malignancies after full COVID‐19 vaccination was evaluated in a meta‐analysis of 49 studies involving 11,086 individuals (PROSPERO study).^55^ The analysis estimated a pooled response of 64% (95% CI 59–69) for hematologic malignancies, compared with 96% (95% CI 92–97) for solid cancers, and 98% (95% CI 96–99) for healthy controls (*p* < 0.001). Specific malignancies had different pooled responses (91% (95% CI 82–96) for HL, 58% [95% CI 44–70] for aggressive NHL, and 61% [95% CI 48–72] for indolent NHL). Disease remission and prior COVID‐19 correlated with higher seroconversion rates, while active treatment was associated with a pooled antibody response of 35%.^55^

While there is accumulating evidence that the humoral immune response to vaccination is impaired in lymphoma patients on B‐cell depleting regimens, the effects of antineoplastic treatment on the cellular immune response have just begun to be addressed. A study in 80 patients with lymphoma who had been treated with anti‐CD20 therapies investigated the humoral and cellular responses after two doses of COVID‐19 vaccine (mostly mRNA‐based) and reported a serum conversion rate of 41%.^53^ Consistent with the findings from other studies, the time from the last anti‐CD20 treatment to COVID‐19 vaccination correlated positively with the rate of seroconversion. T‐cell responses were investigated using two overlapping pools of peptides covering the entire SARS‐CoV‐2 spike protein. Fifty‐eight percent of lymphoma patients and 71% of healthy controls showed a T‐cell response, which was not reduced in patients treated with anti‐CD20 therapy. Notably, 50% of the patients with no seroconversion did show a T‐cell response, suggesting that vaccination may still provide some protection to patients with an insufficient humoral response.^53^

In a recent study in 270 patients with hematologic malignancies who underwent the full cycle of vaccination with the mRNA‐1273 vaccine, the rates of humoral and cellular response were, respectively, 76.3% and 79.0%.^56^ Anti‐CD20 therapy during the past 6 months was associated with a markedly reduced humoral response, but did not substantially affect the cellular response. Whether the observed T‐cell response can prevent a severe/critical course of COVID‐19 needs to be investigated in larger studies.

### 4.3 Booster vaccination

The efficacy of booster COVID‐19 vaccination in lymphoma patients also needs to be addressed. Evidence from studies in cancer patients suggests that those who did not achieve seroconversion after complete vaccination may benefit from an additional dose of vaccine.^34^, ^57^, ^58^ A study involving 88 vaccinated cancer patients, showed that 56% of the 32 patients who were seronegative (mostly patients with hematologic malignancies) achieved seroconversion after booster vaccination.^57^ CAPTURE is a prospective cohort study investigating vaccine response in patients with cancer after two doses of either the BNT162b2 or ChAdOx1vaccine, and after a third vaccination (booster) with BNT162b2.^58^ Data from this study have shown that a third dose of vaccine enhances SARS‐CoV‐2 neutralizing antibodies also in patients who had undetectable neutralizing antibodies after vaccination or demonstrated a waning response.^58^ A study based on the Leukemia & Lymphoma Society US National Patient Registry evaluated serologic responses to a booster dose (given between June‐July 2021) in 49 patients with B‐cell malignancies who had previously received full COVID‐19 vaccination.^59^ Before the booster vaccination, 78% of patients were seronegative. In 32 patients (65%), booster vaccination resulted in an increase in antibody level; these patients were either seroconverted (21 patients) or had increased antibody titers (11 patients). However, 17 patients (35%) continued to be non‐responder despite the booster dose.

A study in 200 patients with lymphoid malignancies evaluated humoral responses after two or three doses of COVID‐19 mRNA vaccine and found significant increases in seroconversion rates and antibody level after the booster dose.^60^ However, benefits were variable across cancer types with <10% of patients with B‐cell malignancies showing seroconversion after the booster dose. A recent analysis of the CAPTURE cohort showed that most fully vaccinated (two doses) cancer patients had no detectable antibodies against the omicron variant of SARS‐CoV‐2, regardless of vaccine type.^61^ Interestingly, a third dose of BNT162b2 led to a significant increase in the number of patients with neutralizing antibodies against the omicron variant; however, among the subgroup of patients with hematologic malignancies, the proportion of those with no detectable neutralizing antibodies continued to be substantial.^58^ Taken together, these preliminary findings indicate that additional doses of COVID‐19 vaccine may be requested in lymphoma patients to improve their humoral response to vaccination. As a consequence, several healthcare systems worldwide are currently offering booster vaccine doses to cancer patients.^61^, ^62^ For example, the Italian Healthcare System has recommended since February 2022 a booster dose of mRNA vaccine (fourth dose) for vulnerable immunosuppressed subjects who have received a full vaccination cycle (two standard vaccine doses and a third dose at ≥28 days from the second dose), to be given after ≥120 days from the third dose.^62^ Indeed, data with hematologic malignancies indicate an immune response after COVID‐19, although lower than that expected in the healthy population. As vaccination after infection strengthens protection and reduces further the risk of re‐infection, immunocompromized patients who recovered from COVID‐19 should undergo vaccination if additional doses are recommended. The benefits of vaccination and boosters are deemed to outweigh the potential and unknown risks for the recipient. We do not support doses of COVID‐19 vaccine beyond those recommended by local guidelines. The time of vaccine should not interfere with the treatment plan for the disease. We recommend for daily clinical practice to vaccine patients before starting therapy (if clinically possible). In the case of a patient on therapy with a long‐lasting immunosuppressive agent (i.e., anti‐CD20 therapies), vaccination should be done anytime.

## 5 PROPHYLAXIS AND THERAPEUTIC OPTIONS FOR SUBJECTS AT HIGH RISK OF COVID‐19 PROGRESSION

Passive immunization with monoclonal antibodies against SARS‐CoV‐2 is an available strategy complementary to vaccination for patients who are unlikely to generate neutralizing antibodies. Antiviral drugs and monoclonal antibodies are valid treatment options for preventing the development of severe‐critical COVID‐19 in vulnerable patients with mild‐moderate disease.

### 5.1 Monoclonal antibodies

Monoclonal antibodies are derived from antibodies isolated from persons infected with SARS‐CoV‐2.^63^ They are administered via intravenous infusion or intramuscular injection, confer protection of variable length, and are currently used for pre‐exposure prophylaxis, post‐exposure prophylaxis, and treatment of mild‐moderate COVID‐19 in subjects at high risk of disease progression. Currently different monoclonal antibodies products are approved by the European Medicines Agency (EMA), for the prevention of COVID‐19 (casirivimab‐imdevimab, tixagevimab‐cilgavimab) or for the treatment of patients with mild‐moderate COVID‐19 at risk of progression to severe disease (bamlanivimab‐etesevimab, casirivimab‐imdevimab, sotrovimab).^64^, ^65^, ^66^, ^67^ The combination bamlanivimab‐etesevimab, in use since March 2021, is no more available due to the low susceptibility profile of the omicron variants of concern (VOC) and on November 2021 EMA has ended the rolling review, after the company informed the Agency that it was withdrawing from the process. The combination casirivimab‐imdevimab contains two recombinant human IgG antibodies that bind non‐competitively to two distinct epitopes of the receptor binding domain of the SARS‐CoV‐2 spike protein.^64^ Sotrovimab neutralizes SARS‐CoV‐2 by targeting an evolutionary conserved epitope outside the rapidly evolving receptor binding domain of the spike protein. Like casirivimab‐imdevimab, the combination tixagevimab‐cilgavimab binds to distinct, non‐overlapping epitopes of the receptor‐binding domain of the spike protein. Phase 3, placebo‐controlled clinical trials in out‐patients with symptomatic COVID‐19 demonstrated significant reductions in hospitalization and mortality in patients who received casirivimab‐imdevimab or sotrovimab versus placebo, with relative risk reductions ranging from 70% to 85%.^26^, ^68^ As for the safety of monoclonal antibodies, no particular safety issues were reported in the pivotal trials, which consistently showed no differences in the adverse event profile of active treatment and placebo.^26^, ^27^, ^28^, ^30^, ^68^

The phase 3 PROVENT trial in patients with an increased risk of responding inadequately to vaccination, an increased risk of exposure to SARS‐CoV‐2, or both, found that patients receiving tixagevimab (150 mg)‐cilgavimab (150 mg) had a 76.7% relative risk reduction of developing symptomatic COVID‐19 versus patients receiving placebo.^30^ Tixagevimab‐cilgavimab is currently approved in Europe for pre‐exposure prophylaxis and for early treatment of SARS‐CoV‐2‐infected patients at high‐risk of disease progression/severity.^69^, ^70^ A recent study in patients with hematologic malignancies showed that tixagevimab (300 mg)‐cilgavimab (300 mg) was able to neutralize the omicron 1 variant of SARS‐CoV‐2.^71^ In consideration of diminished activity against the omicron variants, the FDA granted tixagevimab (300 mg)‐cilgavimab (300 mg) emergency use authorization for pre‐exposure prophylaxis in February 2022, with the possibility of repeated administration every 6 months.^72^ On the contrary, EMA approved the original 150 mg + 150 mg dose, stating that there are no safety and efficacy data available on repeat dosing.^65^

Recently, the ACTIV‐3 trial demonstrated that tixagevimab/cilgavimab used in inpatients with COVID‐19 did not improve recovery time but did reduce deaths,^73^ while TACLE trial showed benefit of tixagevimab/cilgavimab in prevention of severe COVID‐19 in outpatients with mild/moderate infection.^74^

Monoclonal antibodies are limited by the potential loss of efficacy caused by the rapid mutation rate of SARS‐CoV‐2 and the occurrence of new variants that escape the antibody VOC. For example, the combinations bamlanivimab‐etesevimab and casirivimab‐imdevimab are inactive against the currently predominant omicron SARS‐CoV‐2 variant, while sotrovimab and tixagevimab‐cilgavimab have retained some activity.^75^ Bebtelovimab, which was granted an emergency use authorization by the FDA in February 2022 for the treatment of mild‐moderate COVID‐19, is active against all omicron subvariants but it is currently available in US only.^76^ The reader is referred to the following databases for updated information on the activities of current vaccines, monoclonal antibodies and antivirals on VOCs: https://opendata.ncats.nih.gov/variant/activity *and* https://covdb.stanford.edu/susceptibility‐data/table‐mab‐susc/.

### 5.2 Antiviral agents

Currently three antiviral agents are available for the treatment of COVID‐19, namely remdesivir, ritonavir‐boosted nirmatrelvir, and molnupiravir. Remdesivir, a potent inhibitor of the SARS‐CoV‐2 RNA‐dependent RNA polymerase, was first approved by the EMA for the treatment of COVID‐19 in patients with pneumonia requiring oxygen (5‐day therapy), and then also for patients with mild‐moderate COVID‐19 who are at increased risk of progressing to severe COVID‐19.^77^ In this indication it is given by intravenous infusion for 3 days and should be started as soon as possible after diagnosis and within 7 days of symptom onset.^77^ Ritonavir‐boosted nirmatrelvir and molnupiravir are both taken orally and should be started within 5 days from the onset of symptoms. Ritonavir‐boosted nirmatrelvir is approved by the EMA for the treatment of patients with mild‐moderate COVID‐19 at high risk of progressing to severe disease.^78^ Nirmatrelvir inhibits the SARS‐CoV‐2 main protease, which ultimately results in the prevention of viral replication.^78^ Two 150‐mg nirmatrelvir tablets and one 100‐mg ritonavir tablet should be taken twice daily for 5 days; in patients with moderate renal impairment (eGFR ≥30 to < 60 ml/min) the dose should be reduced to one daily tablet of each agent.^78^ Like remdesivir, ritonavir‐boosted nirmatrelvir is not recommended for patients with severe renal impairment (eGFR <30 ml/min).^78^ Molnupiravir received emergency use authorization in December 2021 by the US FDA for the treatment of adults with mild‐moderate COVID‐19 who are at high risk for progression to severe disease^79^; its approval by the EMA is still pending, nevertheless, for such indication, it is already available in several European countries.^80^, ^81^ Molnupiravir is a small‐molecule ribonucleoside prodrug that blocks the replication of SARS‐CoV‐2 and other RNA viruses, by introducing errors in the virus genome. It is taken orally (four 200‐mg capsules every 12 h), for no longer than 5 consecutive days, as soon as possible after COVID‐19 diagnosis.^82^ No dose adjustments are required in case of renal or hepatic impairment.

The efficacy and safety of the three antiviral agents have been demonstrated in large phase 3 randomized, placebo‐controlled trials involving unvaccinated, non‐hospitalized symptomatic patients at high risk for disease progression.^31^, ^32^, ^33^ A short course of remdesivir and ritonavir‐boosted nirmatrelvir was associated with a >85% lower risk of hospitalization or death than placebo.^31^, ^32^ However, virological and clinical rebound has been reported few days after ending ritonavir‐boosted nirmatrelvir full course.^83^ With molnupiravir, the risk of hospitalization or death through day 29 was approximately 50% lower (28 of 385 participants, 7.3%) than with placebo (53 of 377, 14.1%), with a difference in percentage points of −6.8 (95% confidence interval, −11.3 to −2.4, *p* = 0.001) in the interim analysis, while the benefit was lower if all randomized participants were included (6.8% [48 of 709] vs. 9.7% [68 of 699]).^33^ All antiviral agents had an acceptable safety profile in the pivotal trials.^31^, ^32^, ^33^

Antiviral agents have several practical advantages over monoclonal antibodies, including ease of administration and availability. In addition, as they target highly conserved mechanisms, like RNA‐dependent RNA polymerase, they are more likely than monoclonal antibodies targeting the spike protein to retain activity against emerging SARS‐CoV‐2 variants. Preliminary evidence suggests that the omicron SARS‐CoV‐2 variant has a susceptibility to the three antiviral agents similar to that of the original SARS‐CoV‐2 strain.^84^ Limitations of antiviral agents include drug interactions and their potential contribution to the emergence of new viral variants. Ritonavir is a strong inhibitor of the drug‐metabolizing enzyme cytochrome P450 3A; it also has high affinity for P‐glycoprotein and may inhibit this transporter.^78^ Therefore, when prescribing ritonavir‐boosted nirmatrelvir, a careful review of the drugs currently taken by the patients is required. Useful drug interaction resources are available online, for example, the COVID‐19 Drug Interactions checker by the University of Liverpool available at https://covid19‐druginteractions.org/checker. Concerns about the mutagenic potential of molnupiravir and the possibility that it may generate new SARS‐CoV‐2 variants, especially if used in subclinical doses, have been raised.^85^

## 6 PRACTICAL RECOMMENDATIONS FOR PROTECTING LYMPHOMA PATIENTS FROM SARS‐CoV‐2 AND FOR PREVENTING SEVERE/CRITICAL COVID‐19

Under the guidance of the European Society for Medical Oncology (ESMO) and the European Hematology Association (EHA), an interdisciplinary group of experts has recently published a series of consensus statements about the management of patients with hematologic malignancies in the setting of the COVID‐19 pandemic.^24^ The ESMO/EHA statements point out that patients with hematologic malignancies continue to be a vulnerable group and preventive measure including hand washing, mask wearing, and physical distancing should be still implemented; their family members and caregivers should be vaccinated against COVID‐19. COVID‐19 vaccination is strongly recommended also for patients with hematologic malignancies and should be given before starting antineoplastic treatment.^24^ If treatment is urgently needed, however, it should not be postponed because of the vaccination. Vaccines can be safely administered during treatment; however, B‐cell depleting therapies strongly reduce the immune response.^24^ For this reason, seroconversion should be tested in lymphoma patients following vaccination and protective measures should be maintained. Patients presenting COVID‐19 symptoms should be tested with no delay to rapidly confirm the diagnosis and initiate as soon as possible COVID‐19 treatment if indicated. The assessment of seroconversion is recommended also after the exposure to SARS‐CoV‐2, as patients with hematologic malignancies have lower seroconversion rates than the general population also following natural immunization.^42^ With regard to the management of lymphoma, the ESMO/EHA guidelines distinguish between indolent and aggressive lymphoma. When indicated, antineoplastic treatment should be given as recommended, “without compromising efficacy of treatment”.^24^ At the same time, whenever possible, unvaccinated patients should receive COVID‐19 vaccination (at least the first dose) before initiating antineoplastic treatment. In case of newly diagnosed or relapsing aggressive lymphoma, however, treatment has the priority over COVID‐19 vaccination and should not be postponed.^24^

Table 2 summarizes a set of practical recommendations for the management of patients with lymphoma in the current situation of ongoing COVID‐19 pandemic. Three settings of lymphoma management are recognized: diagnosis, which is usually a pretreatment period of watchful waiting; treatment, usually chemotherapy (induction and maintenance) with or without the addition of anti‐CD20 therapy, T‐cell depleting agents, or immunomodulators; follow‐up, a posttreatment period characterized by disease remission. All patients considered in this table should be vaccinated against SARS‐CoV‐2 regardless of the setting of lymphoma management and should be made aware of the latest recommendations about vaccination and booster vaccination recommended for immunocompromized subjects. Owing to the reduced response to vaccines, seroconversion (antibody levels) can be assessed 3–4 weeks after vaccination for scientific purpose but without any impact on vaccination plan or booster doses. Pre‐exposure prophylaxis with tixagevimab‐cilgavimab is recommended in SARS‐CoV‐2 unexposed patients newly diagnosed with lymphoma and in those on active treatment. Recently, tixagevimab‐cilgavimab treatment has been suggested for infected patients at high‐risk of disease progression/severity.^70^ In lymphoma patients exposed to SARS‐CoV‐2, post‐exposure prophylaxis with monoclonal antibodies can be considered if active agents are available (at the time of manuscript writing, monoclonal antibodies approved for post‐exposure prophylaxis were not active against the circulating variants). In general, we recommend the temporary interruption of any procedure and treatment related to lymphoma management in patients who are diagnosed with COVID‐19. However, in patients with mild‐moderate COVID‐19, who are usually outpatients, the decision to interrupt an effective, or urgently needed, antineoplastic treatment should be made on a case‐by‐case basis. In alternative to the discontinuation of the entire antineoplastic treatment regimen, the removal of the B‐cell depleting component could be considered in patients treated with chemotherapy plus anti‐CD20 therapy. For the treatment of COVID‐19, the reader is referred to the latest national/international guidelines.^90^ Currently approved treatment options for mild‐moderate COVID‐19 in lymphoma patients include: monoclonal antibodies, as long as they are active against the circulating virus variants, and antiviral agents to be taken as soon as possible following symptom onset, for 5 consecutive days orally (nirmatrelvir‐ritonavir and molnupiravir) or for 3 consecutive days intravenously (remdesivir).

**TABLE 2:** Practical recommendations for the management of patients with lymphoma and COVID‐19

COVID‐19 disease status						
		Unexposed	Exposed	With mild/moderate COVID‐19 (outpatient)	With severe/critical COVID‐19 (hospitalized)	With history of COVID‐19
Setting of lymphoma management	Diagnosis	Protect from exposure Vaccinate^a^ Start pre‐exposure or primary prophylaxis with tixagevimab‐cilgavimab^b^, if available	SARS‐CoV‐2 testing^c^ Start post‐exposure prophylaxis with monoclonal antibodies, if available	Hold any procedure until resolution of COVID‐19 signs and symptoms and negative SARS‐CoV‐2 test If antineoplastic treatment is urgently needed (aggressive lymphoma), consider chemotherapy alone Start COVID‐19 treatment^d^ as early as possible	Hold any procedure until resolution of COVID‐19 signs and symptoms and negative SARS‐CoV‐2 antigen test Treat COVID‐19 and COVID‐19‐related organ failure^d^	Vaccinate^a^ if > 120 days elapsed from COVID‐19 resolution Start prophylaxis with tixagevimab‐cilgavimab^b^ as soon as possible
	Treatment or ≤6 months since treatment completion	Protect from exposure Vaccinate^a^ Start primary prophylaxis with tixagevimab‐cilgavimab^b^	SARS‐CoV‐2 testing^c^ Start post‐exposure prophylaxis with monoclonal antibodies, if available	Hold antineoplastic treatment until resolution of COVID‐19 signs and symptoms and negative test for SARS‐CoV‐2 Consider to stop anti‐CD20 therapy If antineoplastic treatment is urgently needed (aggressive lymphoma), consider chemotherapy alone Start COVID‐19 treatment^d^ as early as possible	Hold antineoplastic treatment until resolution of COVID‐19 signs and symptoms and negative antigen test for SARS‐CoV‐2 Stop anti‐CD20 therapy Treat COVID‐19 and COVID‐19‐related organ failure^d^	Vaccinate^a^ if > 120 days elapsed from COVID‐19 resolution Start prophylaxis with tixagevimab‐cilgavimab^b^ as soon as possible, if available
	Follow‐up	Protect from exposure Vaccinate^a^	SARS‐CoV‐2 testing	Start COVID‐19 treatment^d^ as early as possible	Treat COVID‐19 and COVID‐19‐related organ failure^d^	Vaccinate^a^ if > 120 days elapsed from COVID‐19 resolution

^a^ According to the latest national/international recommendations for vaccination and for booster doses in immunocompromized subjects (e.g.,: SIE‐GITMO guideline 2021)^86^.

^b^ Treatment should be administered according to tixagevimab‐cilgavimab label^65^.

^c^ According to the latest national/international guidelines^87^.

^d^ According to COVID‐19 severity and to the latest national/international recommendations for COVID‐19 treatment, for example,: WHO Living guidance for clinical management of COVID‐19^88^ and NIH^89^.

## 7 FINAL CONSIDERATIONS AND FUTURE PERSPECTIVES

The field of COVID‐19 prevention and management is changing rapidly, making the continuous update of recommendations necessary. This aspect may pose some limitations to the present effort to provide guidance in the management of patients with lymphoma and COVID‐19. Despite considerable advances in reducing COVID‐19‐related morbidity and mortality, lymphoma patients continue to have an increased risk of developing severe COVID‐19 and to die following the infection. The concomitance of lymphoma and SARS‐CoV‐2 infection complicates the management of both conditions and continues to be challenging for many clinicians. We believe therefore that any attempt to address this challenge may be important to increase the awareness about vulnerable subgroups of patients. The evidence suggests that the response to vaccination can be improved in immunocompromized patients with personalized protocols and repeated booster doses. Monoclonal antibodies to SARS‐CoV‐2 (provided they are active against the circulating variants) and antiviral agents can be used in patients with lymphoma to prevent the progression to severe‐critical COVID‐19. Combined antiviral and monoclonal antibodies strategies are commonly used in real life and the use of combined regimens in lymphoma patients is likely to be an innovative research strategy.^91^ Furthermore, the combination of monoclonal antibodies tixagevimab‐cilgavimab can be used for pre‐exposure prophylaxis.

For daily clinical practice, given the availability of vaccination, tixagevimab‐cilgavimab as pre‐exposure prophylaxis, and antivirals and/or monoclonal antibodies, we recommend to maintain the indication for less immunosuppressive anti‐CD20 agents for follicular lymphomas, as local practice in the pre‐pandemic era.

The benefits of these strategies need to be further evaluated. To this purpose, the collection of real‐world data documenting the use monoclonal antibodies and antiviral agents in lymphoma patients in clinical practice should be encouraged.

## AUTHOR CONTRIBUTIONS

All the authors conceived, performed literature search, selected manuscripts of interest, wrote and edited the manuscript. All the authors approved the final submitted version of the manuscript.

## CONFLICT OF INTEREST

Emanuele Nicastri declares to have received fees from Gilead Science, Eli Lilly, Sobi, Roche; Stefano Luminari declares fees from Roche, Janssen, Gilead/Kite, BMS/Celgene, Regeneron, Genmab; Francesco Passamonti declares honoraria for lecture and advisory boards from Novartis, Celgene, Bristol‐Myers Squibb, Abbvie, Janssen, Roche, AOP Orphan, Karyopharm, Kyowa Kirin and MEI; Grant for research project from BMS. Other authors have nothing to declare.

### PEER REVIEW

The peer review history for this article is available at https://publons.com/publon/10.1002/hon.3086.

## Data Availability

Data sharing not applicable to this article as no datasets were generated or analyzed during the current study.

## References

[1] WHO . Living Guidance for Clinical Management of COVID‐19. Accessed 15 July 2022. https://www.who.int/publications/i/item/WHO‐2019‐nCoV‐clinical‐2021‐2

[2] Williamson EJ , Walker AJ , Bhaskaran K , et al. Factors associated with COVID‐19‐related death using OpenSAFELY. Nature. 2020;584(7821):430‐436. 10.1038/s41586-020-2521-4 32640463 PMC7611074

[3] Liang W , Guan W , Chen R , et al. Cancer patients in SARS‐CoV‐2 infection: a nationwide analysis in China. Lancet Oncol. 2020;21(3):335‐337. 10.1016/S1470-2045(20)30096-6 32066541 PMC7159000

[4] He W , Chen L , Chen L , et al. COVID‐19 in persons with haematological cancers. Leukemia. 2020;34(6):1637‐1645. 10.1038/s41375-020-0836-7 32332856 PMC7180672

[5] Kuderer NM , Choueiri TK , Shah DP , et al. Clinical impact of COVID‐19 on patients with cancer (CCC19): a cohort study. Lancet. 2020;395(10241):1907‐1918. 10.1016/S0140-6736(20)31187-9 32473681 PMC7255743

[6] Passamonti F , Cattaneo C , Arcaini L , et al. Clinical characteristics and risk factors associated with COVID‐19 severity in patients with haematological malignancies in Italy: a retrospective, multicentre, cohort study. Lancet Haematol. 2020;7(10):e737‐e745. 10.1016/S2352-3026(20)30251-9 32798473 PMC7426107

[7] Yigenoglu TN , Ata N , Altuntas F , et al. The outcome of COVID‐19 in patients with hematological malignancy. J Med Virol. 2021;93(2):1099‐1104. 10.1002/jmv.26404 32776581 PMC7436524

[8] Grivas P , Khaki AR , Wise‐Draper TM , et al. Association of clinical factors and recent anticancer therapy with COVID‐19 severity among patients with cancer: a report from the COVID‐19 and Cancer Consortium. Ann Oncol. 2021;32(6):787‐800. 10.1016/j.annonc.2021.02.024 33746047 PMC7972830

[9] Russell B , Moss CL , Shah V , et al. Risk of COVID‐19 death in cancer patients: an analysis from Guy's Cancer Centre and King's College Hospital in London. Br J Cancer. 2021;125(7):939‐947. 10.1038/s41416-021-01500-z 34400804 PMC8366163

[10] Wang Q , Berger NA , Xu R . When hematologic malignancies meet COVID‐19 in the United States: infections, death and disparities. Blood Rev. 2021;47:100775. 10.1016/j.blre.2020.100775 33187811 PMC7833659

[11] El‐Sharkawi D , Iyengar S . Haematological cancers and the risk of severe COVID‐19: exploration and critical evaluation of the evidence to date. Br J Haematol. 2020;190(3):336‐345. 10.1111/bjh.16956 32559308 PMC7323194

[12] Lee LYW , Cazier JB , Starkey T , et al. COVID‐19 prevalence and mortality in patients with cancer and the effect of primary tumour subtype and patient demographics: a prospective cohort study. Lancet Oncol. 2020;21(10):1309‐1316. 10.1016/S1470-2045(20)30442-3 32853557 PMC7444972

[13] Nicastro E , Verdoni L , Bettini LR , et al. COVID‐19 in immunosuppressed children. Front Pediatr. 2021;9:629240. 10.3389/fped.2021.629240 33996683 PMC8116542

[14] Vijenthira A , Gong IY , Fox TA , et al. Outcomes of patients with hematologic malignancies and COVID‐19: a systematic review and meta‐analysis of 3377 patients. Blood. 2020;136(25):2881‐2892. 10.1182/blood.2020008824 33113551 PMC7746126

[15] Armitage JO , Gascoyne RD , Lunning MA , Cavalli F . Non‐Hodgkin lymphoma. Lancet. 2017;390(10091):298‐310. 10.1016/S0140-6736(16)32407-2 28153383

[16] Lewis WD , Lilly S , Jones KL . Lymphoma: diagnosis and treatment. Am Fam Physician. 2020;101(1):34‐41. 31894937

[17] Polack FP , Thomas SJ , Kitchin N , et al. Safety and efficacy of the BNT162b2 mRNA covid‐19 vaccine. N Engl J Med. 2020;383(27):2603‐2615. 10.1056/NEJMoa2034577 33301246 PMC7745181

[18] Baden LR , El Sahly HM , Essink B , et al. Efficacy and safety of the mRNA‐1273 SARS‐CoV‐2 vaccine. N Engl J Med. 2021;384(5):403‐416. 10.1056/NEJMoa2035389 33378609 PMC7787219

[19] Voysey M , Clemens SAC , Madhi SA , et al. Safety and efficacy of the ChAdOx1 nCoV‐19 vaccine (AZD1222) against SARS‐CoV‐2: an interim analysis of four randomised controlled trials in Brazil, South Africa, and the UK. Lancet. 2021;397(10269):99‐111. 10.1016/S0140-6736(20)32661-1 33306989 PMC7723445

[20] Sadoff J , Gray G , Vandebosch A , et al. Safety and efficacy of single‐dose Ad26.COV2.S vaccine against covid‐19. N Engl J Med. 2021;384(23):2187‐2201. 10.1056/NEJMoa2101544 33882225 PMC8220996

[21] Fendler A , de Vries EGE , GeurtsvanKessel CH , et al. COVID‐19 vaccines in patients with cancer: immunogenicity, efficacy and safety. Nat Rev Clin Oncol. 2022;19(6):385‐401. 10.1038/s41571-022-00610-8 35277694 PMC8916486

[22] Sun C , Pleyer C , Wiestner A . COVID‐19 vaccines for patients with haematological conditions. Lancet Haematol. 2021;8(5):e312‐e314. 10.1016/S2352-3026(21)00073-9 33811822 PMC8012057

[23] Gurion R , Rozovski U , Itchaki G , et al. Humoral serological response to the BNT162b2 vaccine is abrogated in lymphoma patients within the first 12 months following treatment with anti‐CD2O antibodies. Haematologica. 2022;107(3):715‐720. 10.3324/haematol.2021.279216 34320790 PMC8883526

[24] Buske C , Dreyling M , Alvarez‐Larran A , et al. Managing hematological cancer patients during the COVID‐19 pandemic: an ESMO‐EHA Interdisciplinary Expert Consensus. ESMO Open. 2022;7(2):100403. 10.1016/j.esmoop.2022.100403 35272130 PMC8795783

[25] Shapiro AE , Bender Ignacio RA . Time to knock monoclonal antibodies off the platform for patients hospitalised with COVID‐19. Lancet Infect Dis. 2022;22(5):567‐569. 10.1016/S1473-3099(21)00762-3 34953521 PMC8700277

[26] Gupta A , Gonzalez‐Rojas Y , Juarez E , et al. Early treatment for covid‐19 with SARS‐CoV‐2 neutralizing antibody sotrovimab. N Engl J Med. 2021;385(21):1941‐1950. 10.1056/NEJMoa2107934 34706189

[27] Gupta A , Gonzalez‐Rojas Y , Juarez E , et al. Effect of sotrovimab on hospitalization or death among high‐risk patients with mild to moderate COVID‐19: a randomized clinical trial. JAMA. 2022;327(13):1236‐1246. 10.1001/jama.2022.2832 35285853 PMC8922199

[28] Weinreich DM , Sivapalasingam S , Norton T , et al. REGN‐COV2, a neutralizing antibody cocktail, in outpatients with covid‐19. N Engl J Med. 2021;384(3):238‐251. 10.1056/NEJMoa2035002 33332778 PMC7781102

[29] Chen P , Nirula A , Heller B , et al. SARS‐CoV‐2 neutralizing antibody LY‐CoV555 in outpatients with covid‐19. N Engl J Med. 2021;384(3):229‐237. 10.1056/NEJMoa2029849 33113295 PMC7646625

[30] Levin MJ , Ustianowski A , De Wit S , et al. Intramuscular AZD7442 (Tixagevimab‐Cilgavimab) for prevention of covid‐19. N Engl J Med. 2022;386(23):2188‐2200. 10.1056/NEJMoa2116620 35443106 PMC9069994

[31] Gottlieb RL , Vaca CE , Paredes R , et al. Early remdesivir to prevent progression to severe covid‐19 in outpatients. N Engl J Med. 2022;386(4):305‐315. 10.1056/NEJMoa2116846 34937145 PMC8757570

[32] Hammond J , Leister‐Tebbe H , Gardner A , et al. Oral nirmatrelvir for high‐risk, nonhospitalized adults with covid‐19. N Engl J Med. 2022;386(15):1397‐1408. 10.1056/NEJMoa2118542 35172054 PMC8908851

[33] Jayk Bernal A , Gomes da Silva MM , Musungaie DB , et al. Molnupiravir for oral treatment of covid‐19 in nonhospitalized patients. N Engl J Med. 2022;386(6):509‐520. 10.1056/NEJMoa2116044 34914868 PMC8693688

[34] Salvini M , Damonte C , Mortara L , et al. Immunogenicity and clinical efficacy of anti‐SARS‐CoV‐2 vaccination in patients with hematological malignancies: results of a prospective cohort study of 365 patients. Am J Hematol. 2022;97(8):E321‐E324. 10.1002/ajh.26629 35702859 PMC9349729

[35] Lamure S , Dulery R , Di Blasi R , et al. Determinants of outcome in Covid‐19 hospitalized patients with lymphoma: a retrospective multicentric cohort study. EClinicalMedicine. 2020;27:100549. 10.1016/j.eclinm.2020.100549 33073216 PMC7550257

[36] Regalado‐Artamendi I , Jimenez‐Ubieto A , Hernandez‐Rivas JA , et al. Risk factors and mortality of COVID‐19 in patients with lymphoma: a multicenter study. Hemasphere. 2021;5(3):e538. 10.1097/HS9.0000000000000538 33604516 PMC7886434

[37] Visco C , Marcheselli L , Mina R , et al. A prognostic model for patients with lymphoma and COVID‐19: a multicentre cohort study. Blood Adv. 2022;6(1):327‐338. 10.1182/bloodadvances.2021005691 34644385 PMC8516438

[38] Bonuomo V , Ferrarini I , Dell'Eva M , Sbisa E , Krampera M , Visco C . COVID‐19 (SARS‐CoV‐2 infection) in lymphoma patients: a review. World J Virol. 2021;10(6):312‐325. 10.5501/wjv.v10.i6.312 34909405 PMC8641038

[39] Garcia‐Suarez J , de la Cruz J , Cedillo A , et al. Impact of hematologic malignancy and type of cancer therapy on COVID‐19 severity and mortality: lessons from a large population‐based registry study. J Hematol Oncol. 2020;13(1):133. 10.1186/s13045-020-00970-7 33032660 PMC7542567

[40] Dulery R , Lamure S , Delord M , et al. Prolonged in‐hospital stay and higher mortality after Covid‐19 among patients with non‐Hodgkin lymphoma treated with B‐cell depleting immunotherapy. Am J Hematol. 2021;96(8):934‐944. 10.1002/ajh.26209 33909916 PMC8212109

[41] Pagano L , Salmanton‐Garcia J , Marchesi F , EPICOVIDEHA working group , et al. COVID‐19 infection in adult patients with hematological malignancies: a European Hematology Association Survey (EPICOVIDEHA). J Hematol Oncol. 2021;14(1):168. 10.1186/s13045-021-01177-0 34649563 PMC8515781

[42] Passamonti F , Romano A , Salvini M , et al. COVID‐19 elicits an impaired antibody response against SARS‐CoV‐2 in patients with haematological malignancies. Br J Haematol. 2021;195(3):371‐377. 10.1111/bjh.17704 34272724 PMC8444788

[43] Schmidt AL , Labaki C , Hsu CY , et al. COVID‐19 vaccination and breakthrough infections in patients with cancer. Ann Oncol. 2022;33(3):340‐346. 10.1016/j.annonc.2021.12.006 34958894 PMC8704021

[44] Heudel P , Favier B , Solodky ML , et al. Survival and risk of COVID‐19 after SARS‐COV‐2 vaccination in a series of 2391 cancer patients. Eur J Cancer. 2022;165:174‐183. 10.1016/j.ejca.2022.01.035 35245864 PMC8828434

[45] Pagano L , Salmanton‐Garcia J , Marchesi F , et al. COVID‐19 in vaccinated adult patients with hematological malignancies: preliminary results from EPICOVIDEHA. Blood. 2022;139(10):1588‐1592. 10.1182/blood.2021014124 34748627 PMC8577877

[46] Pinato DJ , Tabernero J , Bower M , et al. Prevalence and impact of COVID‐19 sequelae on treatment and survival of patients with cancer who recovered from SARS‐CoV‐2 infection: evidence from the OnCovid retrospective, multicentre registry study. Lancet Oncol. 2021;22(12):1669‐1680. 10.1016/S1470-2045(21)00573-8 34741822 PMC8565932

[47] Vijenthira A , Gong I , Betschel SD , Cheung M , Hicks LK . Vaccine response following anti‐CD20 therapy: a systematic review and meta‐analysis of 905 patients. Blood Adv. 2021;5(12):2624‐2643. 10.1182/bloodadvances.2021004629 34152403 PMC8216656

[48] Peeters M , Verbruggen L , Teuwen L , et al. Reduced humoral immune response after BNT162b2 coronavirus disease 2019 messenger RNA vaccination in cancer patients under antineoplastic treatment. ESMO Open. 2021;6(5):100274. 10.1016/j.esmoop.2021.100274 34597941 PMC8423808

[49] Perry C , Luttwak E , Balaban R , et al. Efficacy of the BNT162b2 mRNA COVID‐19 vaccine in patients with B‐cell non‐Hodgkin lymphoma. Blood Adv. 2021;5(16):3053‐3061. 10.1182/bloodadvances.2021005094 34387648 PMC8362658

[50] Jurgens EM , Ketas TJ , Zhao Z , et al. Serologic response to mRNA COVID‐19 vaccination in lymphoma patients. Am J Hematol. 2021;96(11):E410‐E413. 10.1002/ajh.26322 34390501 PMC8420465

[51] Lim SH , Campbell N , Johnson M , et al. Antibody responses after SARS‐CoV‐2 vaccination in patients with lymphoma. Lancet Haematol. 2021;8(8):e542‐e544. 10.1016/S2352-3026(21)00199-X 34224667 PMC8253538

[52] Diefenbach C , Caro J , Koide A , et al. Impaired humoral immunity to SARS‐CoV‐2 vaccination in non‐hodgkin lymphoma and CLL patients. medRxiv. 2021. 10.1101/2021.06.02.21257804

[53] Liebers N , Speer C , Benning L , et al. Humoral and cellular responses after COVID‐19 vaccination in anti‐CD20‐treated lymphoma patients. Blood. 2022;139(1):142‐147. 10.1182/blood.2021013445 34669919 PMC8530768

[54] Sakuraba A , Luna A , Micic D . Serologic response following SARS‐COV2 vaccination in patients with cancer: a systematic review and meta‐analysis. J Hematol Oncol. 2022;15(1):15. 10.1186/s13045-022-01233-3 35123511 PMC8817639

[55] Gagelmann N , Passamonti F , Wolschke C , et al. Antibody response after vaccination against SARS‐CoV‐2 in adults with hematological malignancies: a systematic review and meta‐analysis. Haematologica. 2022;107(8):1840‐1849. 10.3324/haematol.2021.280163 34911284 PMC9335098

[56] Jimenez M , Roldan E , Fernandez‐Naval C , et al. Cellular and humoral immunogenicity of the mRNA‐1273 SARS‐CoV‐2 vaccine in patients with hematologic malignancies. Blood Adv. 2022;6(3):774‐784. 10.1182/bloodadvances.2021006101 34844263 PMC8632354

[57] Shapiro LC , Thakkar A , Campbell ST , et al. Efficacy of booster doses in augmenting waning immune responses to COVID‐19 vaccine in patients with cancer. Cancer Cell. 2022;40(1):3‐5. 10.1016/j.ccell.2021.11.006 34838186 PMC8595142

[58] Fendler A , Shepherd STC , Au L , et al. Immune responses following third COVID‐19 vaccination are reduced in patients with hematological malignancies compared to patients with solid cancer. Cancer Cell. 2022;40(2):114‐116. 10.1016/j.ccell.2021.12.013 34968417 PMC8716090

[59] Greenberger LMLAS , Senefeld JW , Johnson PW , DeGennaro LJ , Nichols GL , Nichols GL . Sars‐Cov‐2 antibody levels in blood cancer patients after a third sars‐cov‐2 “booster” vaccination – observational data from the LLS national registry. Blood. 2021;138(Suppl 1):185. Oral Abstract. 63rd ASH Annual Meeting Abstract. 10.1182/blood-2021-151419

[60] Gressens SB , Fourati S , Le Bouter A , et al. Anti‐SARS‐CoV‐2 antibody response after 2 and 3 doses of BNT162b2 mRNA vaccine in patients with lymphoid malignancies. Clin Microbiol Infect. 2022;28(6):885.e7‐885.e11. 10.1016/j.cmi.2022.02.029 PMC8897197 35259530

[61] Fendler A , Shepherd STC , Au L , et al. Omicron neutralising antibodies after third COVID‐19 vaccine dose in patients with cancer. Lancet. 2022;399(10328):905‐907. 10.1016/S0140-6736(22)00147-7 35090602 PMC8789238

[62] EMA . COVID‐19: Joint Statement from ECDC and EMA on the Administration of a Fourth Dose of mRNA Vaccines. Accessed 18 July 2022. https://bit.ly/3PAQznN

[63] Sullivan DJ , Gebo KA , Shoham S , et al. Early outpatient treatment for covid‐19 with convalescent plasma. N Engl J Med. 2022;386(18):1700‐1711. 10.1056/NEJMoa2119657 35353960 PMC9006786

[64] EMA . Casirivimab‐imdevimab SPC. Accessed 18 July 2022. https://bit.ly/3IOAHw3

[65] EMA . Tixagevimab‐cilgavimab SPC. Accessed 18 July 2022. https://bit.ly/3PH8JEJ

[66] EMA . Bamlanivimab‐etesevimab SPC. Accessed 18 July 2022. https://bit.ly/3Ohg8tb

[67] EMA . Sotrovimab SPC. Accessed 18 July 2022. https://bit.ly/3Ohc9wA

[68] Weinreich DM , Sivapalasingam S , Norton T , et al. REGEN‐COV antibody combination and outcomes in outpatients with covid‐19. N Engl J Med. 2021;385(23):e81. 10.1056/NEJMoa2108163 34587383 PMC8522800

[69] AIFA . Inserimento del medicinale «Evusheld» (associazione di anticorpi monoclonali tixagevimab e cilgavimab) nell'elenco dei medicinali ai sensi della legge 23 dicembre 1996, n. 648. (Determina n. DG/344/2022). Accessed 26 September 2022. https://www.aifa.gov.it/documents/20142/961234/Determina_DG‐344‐2022_Evusheld.pdf

[70] EMA . EMA Recommends Authorisation of COVID‐19 Medicine Evusheld. Accessed 26 September 2022. https://www.ema.europa.eu/en/news/ema‐recommends‐authorisation‐covid‐19‐medicine‐evusheld

[71] Stuver R , Shah GL , Korde NS , et al. Activity of AZD7442 (tixagevimab‐cilgavimab) against Omicron SARS‐CoV‐2 in patients with hematologic malignancies. Cancer Cell. 2022;40(6):590‐591. 10.1016/j.ccell.2022.05.007 35598602 PMC9108069

[72] FDA . FDA Authorizes Revisions to Evusheld Dosing. Accessed 18 July 2022. https://bit.ly/3Ph2MhR

[73] Group AC‐‐TfIwC‐ S , Paredes R , Murray TA , et al. Tixagevimab‐cilgavimab for treatment of patients hospitalised with COVID‐19: a randomised, double‐blind, phase 3 trial. Lancet Respir Med. 2022;10(10):972‐984. 10.1016/S2213-2600(22)00215-6 35817072 PMC9270059

[74] Montgomery H , Hobbs FDR , Padilla F , et al. Efficacy and safety of intramuscular administration of tixagevimab‐cilgavimab for early outpatient treatment of COVID‐19 (TACKLE): a phase 3, randomised, double‐blind, placebo‐controlled trial. Lancet Respir Med. 2022;10(10):985‐996. 10.1016/S2213-2600(22)00180-1 35688164 PMC9173721

[75] Takashita E , Kinoshita N , Yamayoshi S , et al. Efficacy of antibodies and antiviral drugs against covid‐19 omicron variant. N Engl J Med. 2022;386(10):995‐998. 10.1056/NEJMc2119407 35081300 PMC8809508

[76] FDA . Coronavirus (COVID‐19) Update: FDA Authorizes New Monoclonal Antibody for Treatment of COVID‐19 that Retains Activity against Omicron Variant. Accessed 18 July 2022. https://bit.ly/3AVmKKJ

[77] EMA . Remdesivir SPC. Accessed 16 July 2022. https://bit.ly/3Pk203t

[78] EMA . Ritonavir and PF‐07321332 SPC. Accessed 18 July 2022. https://bit.ly/3yKsDHU

[79] FDA . Coronavirus (COVID‐19) Update: FDA Authorizes Additional Oral Antiviral for Treatment of COVID‐19 in Certain Adults. Accessed 18 July 2022. https://bit.ly/3ocGYbh

[80] AIFA . Definizione delle modalita' e delle condizioni di impiego dell'antivirale «Lagevrio» (molnupiravir). (Determina n. DG/1644/2021). (21A07770) (GU Serie Generale n.308 del 29‐12‐2021). Accessed 26 September 2022. https://www.gazzettaufficiale.it/atto/serie_generale/caricaDettaglioAtto/originario?atto.dataPubblicazioneGazzetta=2021‐12‐29%26atto.codiceRedazionale=21A07770%26elenco30giorni=false

[81] ECDC . Treatment and Pharmaceutical Prophylaxis of COVID‐19. Accessed 29 September 2022. https://www.ecdc.europa.eu/en/covid‐19/latest‐evidence/treatment

[82] EMA . Lagevrio (Molnupiravir) SPC. Accessed 18 July 2022. https://bit.ly/3uXFW6E

[83] Antonelli G , Focosi D , Turriziani O , et al. Virological and clinical rebounds of COVID‐19 soon after nirmatrelvir/ritonavir discontinuation. Clin Microbiol Infect. 2022:S1198‐743X(22)00344‐5. 10.1016/j.cmi.2022.06.029 PMC9250152 35792281

[84] Takashita E , Kinoshita N , Yamayoshi S , et al. Efficacy of antiviral agents against the SARS‐CoV‐2 omicron subvariant BA.2. N Engl J Med. 2022;386(15):1475‐1477. 10.1056/NEJMc2201933 35263535 PMC8929374

[85] Service RF .A prominent virologist warns COVID‐19 pill could unleash dangerous mutants. Others see little cause for alarm. Science. 2021. 10.1126/science.acx9591

[86] Società Italiana di Ematologia (SIE) e del Gruppo Italiano Trapianto di Midollo Osseo (GITMO) . Vaccinazione per COVID‐19 nei pazienti con malattie del sangue e sottoposti a trapianto di cellule staminali. Accessed 18 July 2022. https://www.siematologia.it/storage/siematologia/article/pdf/22/2‐documento‐sie‐gitmo_20210210‐124518.pdf

[87] NIH . Testing for SARS‐CoV‐2 Infection. Summary Reccomendation. Accessed 18 July 2022. https://www.covid19treatmentguidelines.nih.gov/overview/sars‐cov‐2‐testing/

[88] WHO . Living Guidance for Clinical Management of COVID‐19. Accessed 18 July 2022. https://www.who.int/publications/i/item/WHO‐2019‐nCoV‐clinical‐2021‐2

[89] NIH . COVID‐19 Treatment Guidelines. Clinical Management Summary. Accessed 18 July 2022. https://www.covid19treatmentguidelines.nih.gov/management/clinical‐management/clinical‐management‐summary/

[90] NIH . Coronavirus Disease 2019 (COVID‐19) Treatment Guidelines. Accessed 16 July 2022. https://www.covid19treatmentguidelines.nih.gov/ 34003615

[91] D'Abramo A , Vita S , Maffongelli G , et al. Clinical management of patients with B‐cell depletion agents to treat or prevent prolonged and severe SARS‐COV‐2 infection: defining a treatment pathway. Front Immunol. 2022;13:911339. 10.3389/fimmu.2022.911339 35711444 PMC9196078

